# Effect of anterior translation of the talus on outcomes of three-component total ankle arthroplasty

**DOI:** 10.1186/1471-2474-14-260

**Published:** 2013-09-05

**Authors:** Keun-Bae Lee, Myung-Sun Kim, Kyung-Soon Park, Kyu-Jin Cho, Andri Primadhi

**Affiliations:** 1 Department of Orthopedic Surgery, Chonnam National University Medical School and Hospital, 42 Jebongro, Donggu, Gwangju 501-757, Republic of Korea; 2 Center for Joint Disease, Chonnam National University Hwasun Hospital, 160 Ilsimri, Hwasun eup, Hwasun gun, Jeonnam 519-763, Republic of Korea; 3 Department of Orthopaedics and Traumatology, Padjadjaran University / Hasan Sadikin Hospital, Bandung, Indonesia

**Keywords:** Osteoarthritis, Total ankle arthroplasty, Three-component prosthesis, Anterior translation of the talus

## Abstract

**Background:**

Ankle osteoarthritis commonly involves sagittal malalignment with anterior translation of the talus relative to the tibia. Total ankle arthroplasty has become an increasingly popular treatment for patients with symptomatic ankle osteoarthritis. However, no comprehensive study has been conducted on the outcomes of total ankle arthroplasty for osteoarthritis with preoperative sagittal malalignment. The purpose of this study was to evaluate the effect of anterior translation of the talus on outcomes of three-component total ankle arthroplasty.

**Methods:**

One hundred and four osteoarthritic ankles in 104 patients who underwent three-component total ankle arthroplasty were included in this study. The 104 ankles were divided into 2 groups: ankles with anteriorly translated talus (50 ankles), and ankles with non-translated talus (54 ankles). Clinical and radiographic outcomes were assessed in both groups. The mean follow-up duration was 42.8 ± 17.9 months (range, 24 to 95 months).

**Results:**

Forty-six (92%) of 50 ankles with anterior translation of the talus showed relocation of the talus within the mortise at 6 months, and 48 (96%) ankles were relocated at 12 months after total ankle arthroplasty. But, 2 (4%) ankles were not relocated until the final follow-up. The AOFAS scores, ankle range of motion, and radiographic outcomes showed no significant difference between the two groups at the final follow-up (p > 0.05 for each).

**Conclusions:**

In majority of cases, the anteriorly translated talus in osteoarthritic ankles was restored to an anatomical position within 6 months after successful three-component total ankle arthroplasty. The clinical and radiographic outcomes in the osteoarthritic ankles with anteriorly translated talus group were comparable with those in non-translated talus group.

## Background

Ankle osteoarthritis often involves ankle sagittal malalignment, including anterior or posterior translation of the talus relative to the tibia, and anterior translation of the talus seems to be more common [1-4]. While there is clinical evidence for anterior translation of the talus in ankle osteoarthritis, the mechanism of anterior translation of the talus is not well understood. According to literature review, the anterior opening angle of the distal tibial joint surface increases in ankle osteoarthritis, loading stress is concentrated on the front of the ankle, and the talus is translated anteriorly due to stretching of the anterior talofibular ligament [5,6].

In recent years, total ankle arthroplasty has become more popular as a treatment option in patients with osteoarthritis of the ankle, with favorable clinical results [1,2,7,8]. Restoring the anatomical orientation of the tibia and talar component is considered vital to good long-term results of total ankle arthroplasty, [7,9,10] and proper positioning of the talar component is another important issue in total ankle arthroplasty [11,12]. Sagittal malalignment in total ankle arthroplasty is one possible cause of premature implant failure, and unfavorable mechanical effects of implant malpositioning in the sagittal plane have been described in cadaver-based experimental studies [13,14].

One study [2] demonstrated that 46 (33%) of 138 osteoarthritic ankles had anterior translation of the talus. Of these 46 ankles, 40 (87%) ankles showed relocation of the talus within the ankle mortise, 5 (11%) ankles showed unchanged alignment of the talus, and 1 ankle showed progression of translation of the talus at the average 54 month follow-up. However, they did not reported outcomes of total ankle arthroplasty for osteoarthritic ankles with and without anterior translation of the talus. Therefore, a study was needed for the evaluation of the rate and the time of the relocation of the anteriorly translated talus into the ankle mortise after total ankle arthroplasty, and the effect of anterior translation of the talus on outcomes of total ankle arthroplasty.

The purpose of the present study was to evaluate the relocation of an anteriorly translated talus into the mortise after total ankle arthroplasty, and the effect of anterior translation of the talus on outcomes of three-component total ankle arthroplasty.

## Methods

This retrospective study was approved by the institutional review board of Chonnam National University Hospital, and informed consent was obtained from all patients. One hundred and twenty consecutive total ankle arthroplasties were performed in 118 patients (120 ankles) with symptomatic ankle osteoarthritis using the cementless three-component HINTEGRA prosthesis (Newdeal, SA, Lyon, France) by a single surgeon, between January 2005 and December 2009.

The inclusion criterion for this study was symptomatic end-stage ankle osteoarthritis with fulfillment of general total ankle arthroplasty indications (good bone stock, normal vascular status, sufficient medial and lateral ankle stability). We excluded 7 patients (9 ankles) because of rheumatoid arthritis in 5 ankles, avascular necrosis of the talus in two ankles, diabetic Charcot arthropathy in one ankle, and revision surgery due to deep infection in one ankle. Three patients (3 ankles) without adequate follow-up for at least 24 months were also excluded.

The remaining 108 osteoarthritic ankles in 108 patients comprised of 50 ankles with anterior translation of the talus (46.3%), 54 ankles with non-translation of the talus (50%), and 4 ankles with posterior translation of the talus (3.7%). Because our objective was to evaluate outcomes of primary total ankle arthroplasty for ankle osteoarthritis with anterior translation of the talus, we excluded these four ankles with posterior translation of the talus.

The study cohort consisted of 104 patients (104 ankles) who had ankle osteoarthritis with a mean follow-up period of 42.8 ± 17.9 months (range, 24 to 95 months). There were 72 men and 32 women with a mean age of 62 years (range, 22 to 77 years). The average body mass index was 25.6 kg/m^2^ (range, 18.8 to 39.3 kg/m^2^). Fifty-six ankles had primary osteoarthritis, and 48 ankles had post-traumatic osteoarthritis. The average preoperative ankle range of motion (dorsiflexion plus plantar flexion of the ankle) was 31 degrees (range, 5 to 60 degrees). One hundred and four osteoarthritic ankles were divided into two groups: Group A (osteoarthritic ankles with an anteriorly translated talus) included 50 ankles and Group B (osteoarthritic ankles with a non-translated talus) included 54 ankles. Preoperative demographic data did not show any significant difference between two groups only without operation time. Osteoarthritis with anterior translation of the talus commonly showed more progressed status of the disease. They needed more cautious approach and were more difficult to make the proper alignment with good soft tissue balance. Those are thought to be the reason of longer operation time of group A than group B (Table 1).

**Table 1:** Comparison of patients with and without anterior translation of the talus

	**Anterior translation of Talus (N = 50)**	**Non-translation of Talus (N = 54)**	P Value*
**Male/Female (no)**	36/14	36/18	0.602
**Age (year) **^†^	64.3 ± 13.4	59.4 ± 8.9	0.133
**Body mass index (kg/m**^**2**^**) **^†^	25.7 ± 3.5	25.5 ± 3.5	0.887
**Preoperative diagnosis**			0.631
**Primary osteoarthritis (no.(%))**	29(58.0)	27(50.0)	
**Posttraumatic osteoarthritis (no.(%))**	21(42.0)	27(50.0)	
**Prior ankle procedure (no.(%))**	8(40.0)	18(29.0)	0.412
**Operation time (minutes) **^†^	124.5 ± 10.0	110.9 ± 11.8	0.000
**Duration of follow-up (month) **^†^	42.2 ± 16.4	43.4 ± 19.5	0.752

* The p values shown are for intergroup comparisons. Significance was accepted for p values of < 0.05.

^†^ The values are given as the mean and the standard deviation.

In a control group of 50 normal ankles in our clinic, the mean tibiotalar ratio (and standard deviation) was 35 ± 3%. If the 95% confidence interval in the control group (29% to 41%) is taken as representative of the normal range for healthy populations, significant translation of the talus was decided outside of the two SDs (Table 2).

**Table 2:** Criteria of anterior translation of the talus in ankle osteoarthritis

**Type**	**Criteria**	**Tibiotalar ratio (%)**
**Control**		35 ± 3*
**Non-translation of Talus**	T-T^†^ ratio ± 2SD	29 ≤ T-T ratio ≤ 41
**Anterior translation of Talus**	T-T ratio < 2SD	T-T ratio < 29

*The values are given as the mean and the standard deviation.

^†^*T-T* Tibiotalar.

### Operative technique and postoperative management

All patients received total ankle arthroplasty using a longitudinal anterior approach between the anterior tibial tendon and extensor hallucis longus with the patient in the supine position. After removal of anterior capsular synovial tissue and osteophytes, the tibial cut was made to spare as much subchondral bone as possible. The superior talar cut was made parallel to the tibial cut, then the medial and lateral talar cuts were made, and finally the posterior cut was performed. The talar component was placed in the anatomical position of the talar dome. The selected implants were inserted after irrigation of the ankle. The wound was closed around the closed suction drain. All patients followed the same postoperative regimen. For the first two weeks after surgery, a well-padded short leg splint was used to keep the foot in a neutral position and to protect the ankle against plantar flexion movement, and no weight-bearing was allowed. For the next two weeks, an ankle-foot orthosis was used, and the patients were mobilized with weight-bearing as tolerated. Full weight-bearing and a foot and ankle rehabilitation program, which included stretching of the triceps surae, calf strengthening, and proprioceptive training were started after four weeks from surgery. Patients were instructed these exercise to perform about 20 minutes in a session and 2 sessions in a day by themselves for 8 weeks. All patients were followed at one, three, six, and twelve months postoperatively, and annually thereafter.

### Clinical evaluation

Clinical outcomes were evaluated by 2 reviewers, who were not directly involved in the surgical procedure, using the American Orthopaedic Foot and Ankle Society (AOFAS) ankle-hindfoot scale [15]. The 100-point AOFAS scoring system combines subjective and objective data to evaluate clinical parameters; pain (40 points), function (45 points), and alignment (15 points). The AOFAS score is the most commonly reported outcome assessment after total ankle arthroplasty, but evidence of its validity and reliability is limited [16]. Ankle range of motion (ROM) was determined clinically using a goniometer along the lateral border of the leg and foot, and radiographically using the angle between talar and tibiar component on the lateral view [17]. We also noted the perioperative complications and additional procedures.

### Radiographic evaluation

Radiographic examination included anteroposterior and lateral radiographs of ankles on full weight-bearing taken preoperatively, at 3 and 6 months postoperatively, and annually thereafter. All radiological measurement were measured accurately using the standard tools in our PACS (Picture Archiving and Communication System: Marotech 5.4) and evaluated by 2 reviewers, who were not directly involved in the surgical procedure.

**Figure 1:**
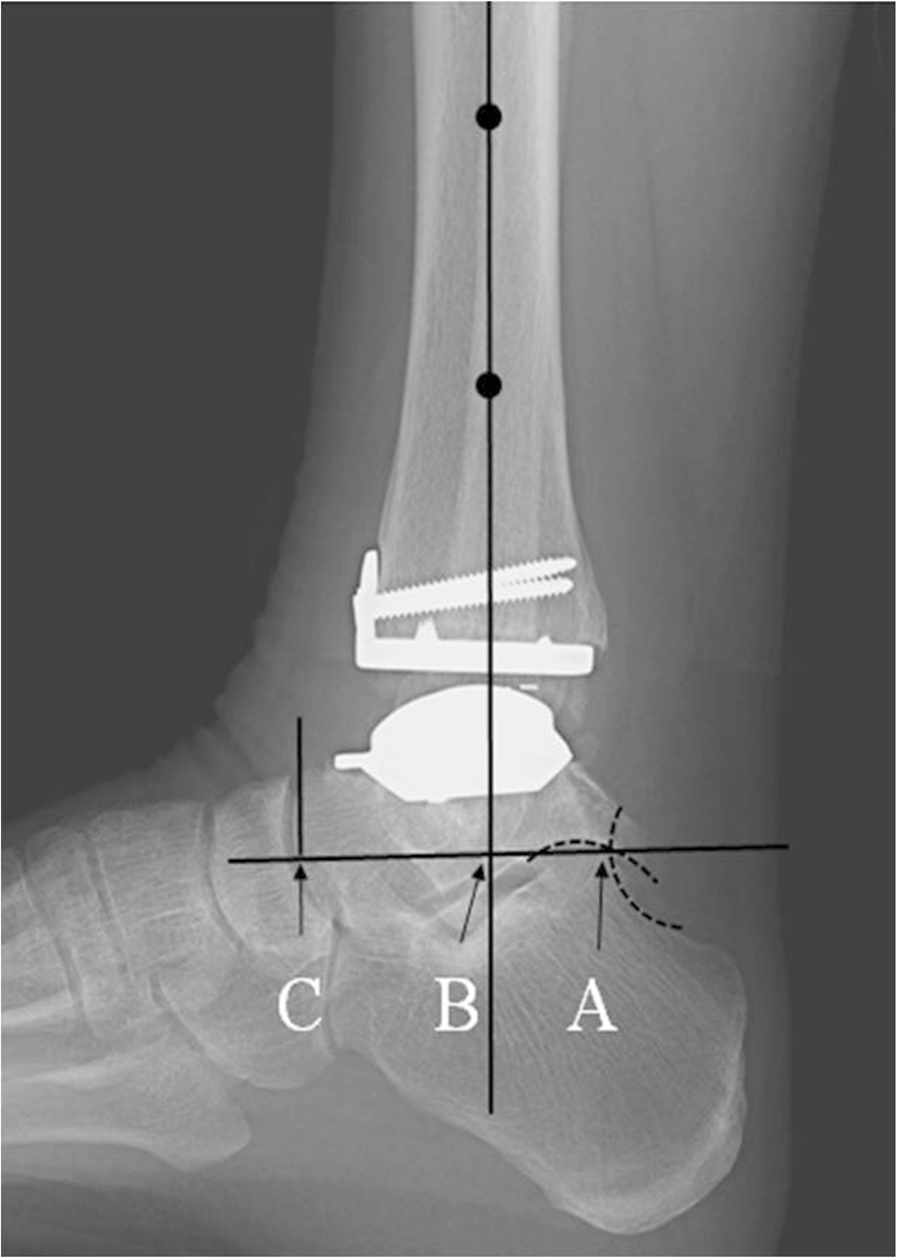
**Lateral radiograph showing the measurements used to determine the postoperative tibiotalar ratio (tibiotalar ratio = AB / AC x 100).** The posterior talar point (point A) was defined as the intersection between the contours of the posterior subtalar articular surface and the posterosuperior calcaneal cortex, and the talar reference line was defined as the line through point A parallel to the floor. The anterior talar point (point C) was defined as the vertical projection of the most anterior aspect of the talus onto the talar reference line. Longitudinal talar length was defined as the length AC, and the distal tibial axis was defined as the longitudinal middisecting line of the distal tibial shaft determined at 5 and 10 cm above the ankle.

**Figure 2:**
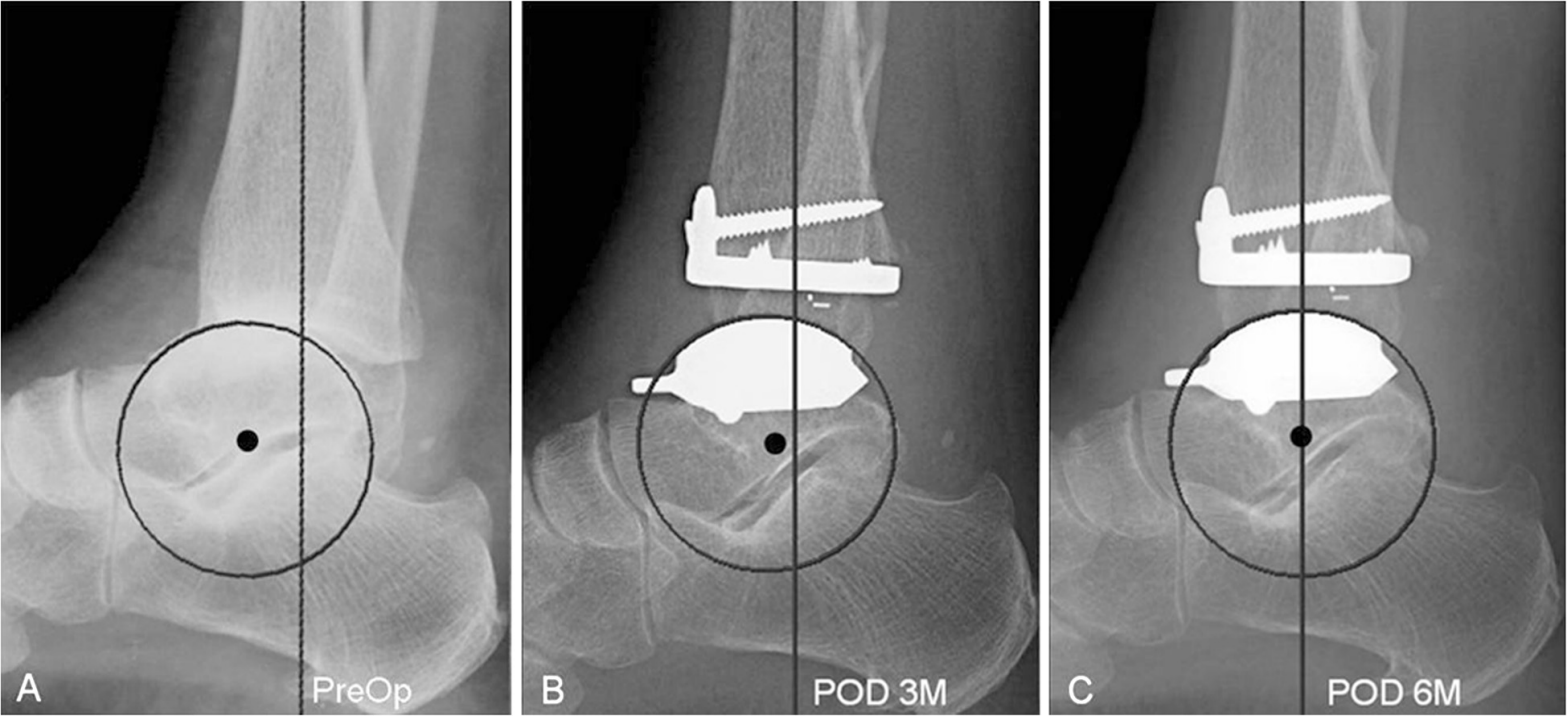
**A 42-year-old woman who underwent primary total ankle arthroplasty (A,B,C) for ankle osteoarthritis. (A)** Preoperative lateral weight bearing radiograph shows anterior translation of the talus. **(B **and **C)** Postoperative lateral weight bearing radiographs show that the anteriorly translated talus was restored to an anatomical position after primary total ankle arthroplasty.

Anteroposterior tibial-talar alignment was quantified using tibiotalar ratio (ratio into which the mid-longitudinal axis of the tibial shaft divides the longitudinal talar length) on lateral ankle radiographs [4,17] (Figure 1). The angular positions of the tibial components were assessed using the α and β angles and the talar component positions were assessed using γ angles [8,17] (Figure 2). Loosening of the tibial component was defined as a change in position of the flat base of the component in relation to the long axis of the tibia by >2° or a progressive radiolucency > 2 mm. Loosening of the talar component was defined as subsidence into the talar bone of >5 mm, or a change in γ angles of >5°.

### Statistical analysis

Descriptive statistics (arithmetic means, averages, and ranges) were calculated using standard formulas. The independent t-test was used to determine the significance of inter-group differences in age, follow-up duration. Mann–Whitney U-test was used to determine the significance of inter-group differences in AOFAS score, ROM, and radiographic values (α, β, γ angles, and tibiotalar ratio) between the two groups. Also, Wilcoxon signed rank test was used to assess the intra-group differences of clinical and radiographic results before and after surgery. The Pearson chi-square test was conducted to determine the significance of inter-group differences in complication incidence, and additional procedure rates. Statistical significance was defined as p <0.05.

## Results

**Figure 3:**
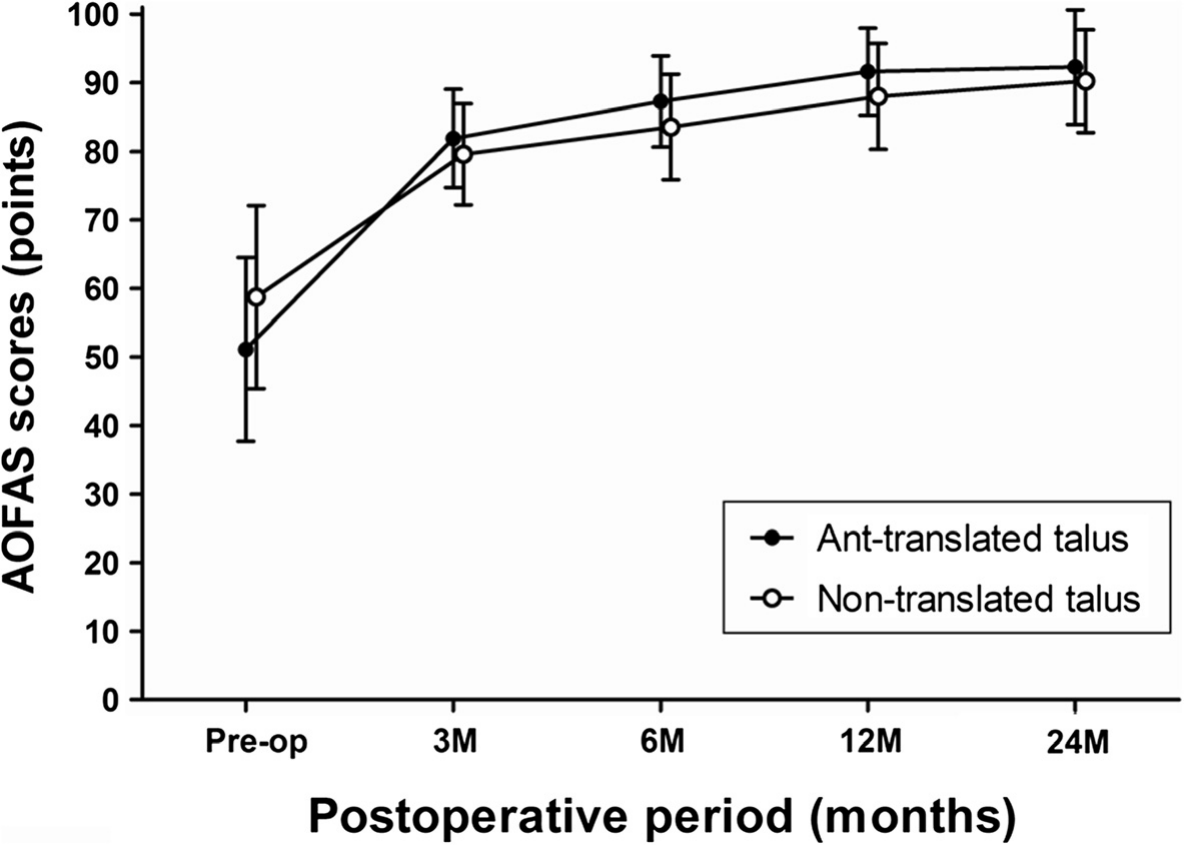
**Time course of the postoperative AOFAS scores for the patients of ankle osteoarthritis with or without an anteriorly translated talus after primary total ankle arthroplasty.** The error bars represent the standard deviation. There were no significant differences at all study periods.

**Figure 4:**
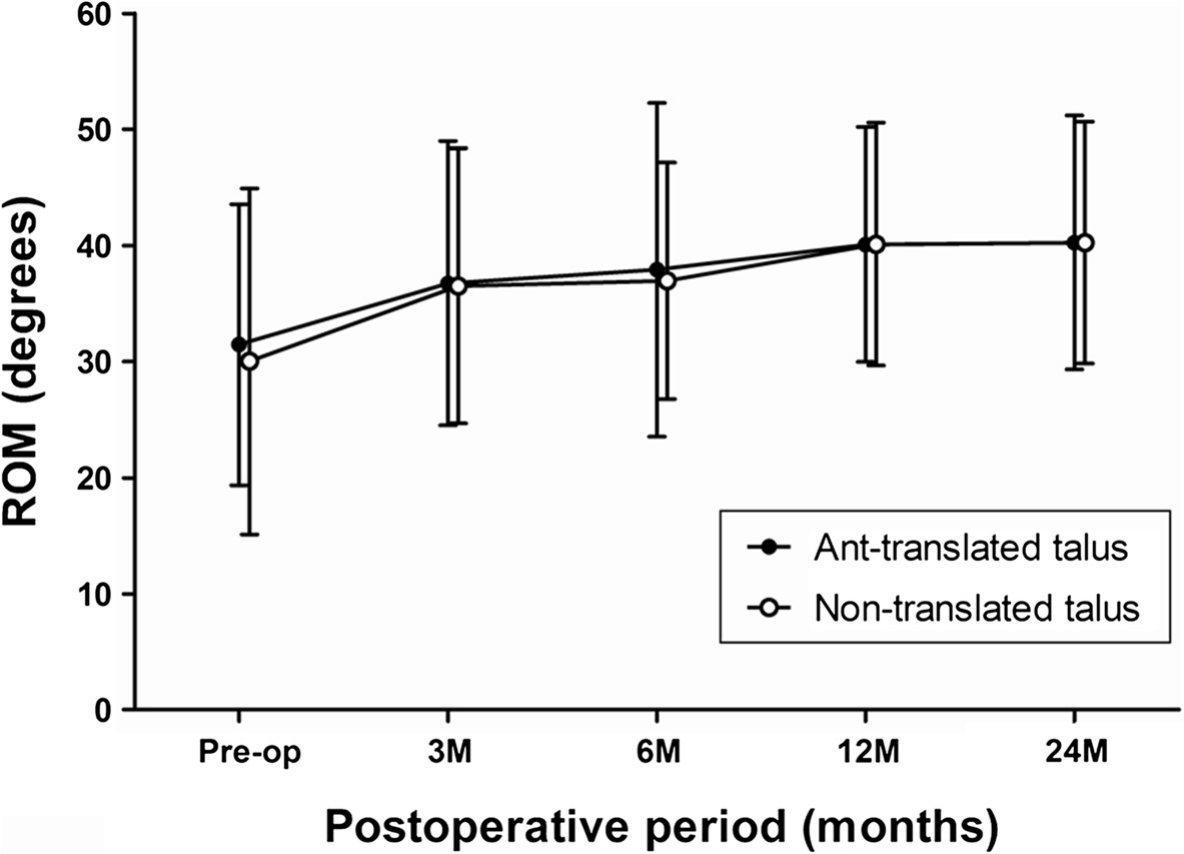
**Time course of the postoperative ankle range of motion in the patients with or without an anteriorly translated talus after primary total ankle arthroplasty.** The error bars represent the standard deviation. There were no significant differences at all study periods.

Mean AOFAS ankle-hindfoot score improved from 46.2 points preoperatively to 93.0 points at the final follow-up in Group A and from 52.5 points preoperatively to 91.0 points at the final follow-up in Group B. There was no significant difference in the AOFAS score during the study period, between the two groups from preoperatively until the final follow-up (Figure 3). Mean ankle range of motion was 28.7 degrees preoperatively and 40.3 degrees at the final follow-up in Group A and 32.5 degrees preoperatively and 39.0 degrees at the final follow-up in Group B. There was also no significant difference in the ROM during the study period, between the 2 groups from preoperative evaluation until the final follow-up (Table 3) (Figure 4).

**Table 3:** Comparison of clinical outcomes of patients with and without anterior translation of the talus

**Clinical Outcomes**	**Anterior translation of Talus**	**Non-translation of Talus**	**P Value**^**†**^
	**(N = 50)**	**(N = 54)**	
AOFAS scores* (points)			
**Preoperative**	46.2 ± 13.4	52.5 ± 11.6	0.085
**Final follow-up**	93.0 ± 6.6	91.0 ± 13.3	0.290
**P Value‡**	0.002	0.043	
**Range of motion (º)**			
**Preoperative**	28.7 ± 12.1	32.5 ± 14.9	0.241
**Final follow-up**	40.3 ± 10.9	39.0 ± 10.4	0.198
**P Value**	0.001	0.032	

The values are given as the mean and the standard deviation.

*AOFAS scores American Orthopaedic Foot and Ankle Society ankle-hindfoot scores.

^**†**^ Mann–Whitney U-test. The *p* values shown are for intergroup comparisons. Significance was accepted for *p* values of < 0.05.

‡Wilcoxon signed rank test. The *p* values pertain to the comparisons between the preoperative and final follow-up examinations. The level of significance was set at *p* values < 0.05.

**Figure 5:**
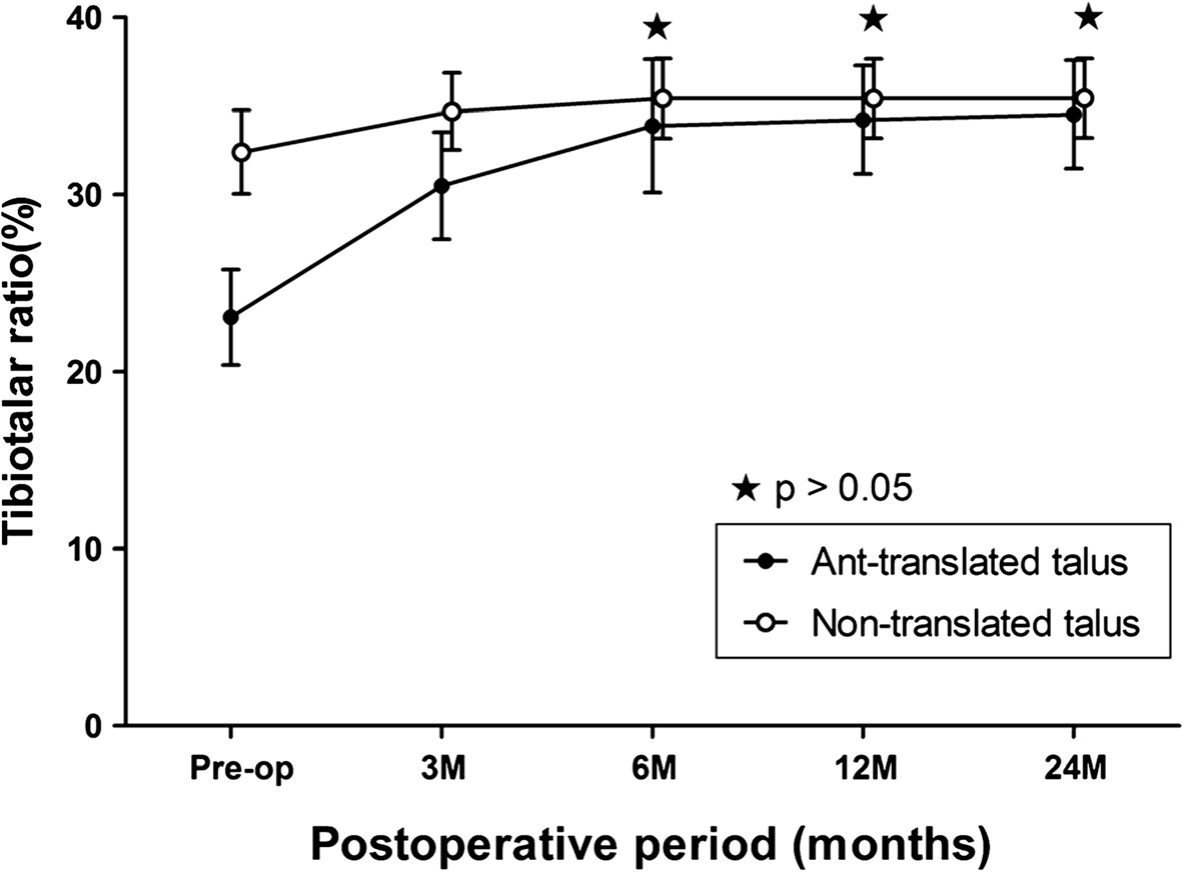
**Time course of the postoperative tibiotalar ratio in the patients with or without an anteriorly translated talus after primary total ankle arthroplasty.** The error bars represent the standard deviation.

Forty-six (92%) of 50 ankles (Group A) with anterior translation of the talus showed relocation of the talus within the mortise at 6 months after primary total ankle arthroplasty, 48 (96%) ankles showed relocation of the talus within the mortise at 12 months, and remained 2 (4%) ankles showed unchanged alignment of the talus until the final follow-up after primary total ankle arthroplasty (Figure 5). The mean tibiotalar ratio in Group A improved from 23.1% preoperatively to 34.5% at the final follow-up. In Group B with non-translation of the talus, the mean tibiotalar ratio improved from 32.4% preoperatively to 35.4% at the final follow-up (Table 4). All ankles of group B had a normal range of tibiotalar ratio postoperatively. During the follow-up period, although the mean tibiotalar ratio of Group A was restored within the normal range (29% to 41%), the mean tibiotalar ratio of Group A had a relatively lower value than that of Group B during the all study period. But, there was no significant difference at 6, 12 months, and at the final follow-up. (p = 0.068, 0.164, 0.368, respectively) (Figure 5).

**Table 4:** Comparison of radiographic outcomes of patients with and without anterior translation of the talus

**Radiographic parameters**	**Anterior translation of**	**Non-translation of**	P Value^*^
	**Talus (N = 50)**	**Talus (N = 54)**	
**Tibiotalar ratio (%)**			
**Preoperative**	23.1 (16.1-27.7)	32.4 (29.0-37.0)	0.000
**Final follow-up**	34.5 (25.1-39.8)	35.4 (31.8-39.5)	0.368
**P Value**^**†**^	0.000	0.000	
**(α) AP**^**‡ **^**tibial bone-component angle (º)**	89.1 (81.5-94.7)	89.2 (84.1-95.4)	0.751
**(β) Lateral tibial bone-component angle (º)**	85.2 (81.3-94.1)	85.4 (79.9-92.6)	0.993
**(γ) Lateral talar bone-component angle (º)**	15.7 (7.8-24.6)	14.8 (3.6-23.2)	0.579

The values are given as the mean, with the range in parentheses.

* Mann–Whitney U-test. The *p* values shown are for intergroup comparisons. Significance was accepted for *p* values of < 0.05.

**†**Wilcoxon signed rank test. The *p* values pertain to the comparisons between the preoperative and final follow-up examinations. The level of significance was set at *p* values < 0.05.

‡ *AP* Anteroposterior.

Measured angles, linear values were not significantly different between the 2 groups. In Group A and Group B, the tibial components were positioned in the coronal plane at an average α angle of 89.1 degrees (range, 81.5 to 94.7 degrees) and 89.2 degrees (range, 84.1 to 95.4 degrees), respectively, and in the sagittal plane at an average β angle of 85.2 degrees (range, 81.3 to 94.1 degrees) and 85.4 degrees (range, 79.9 to 92.6 degrees), respectively. The talar component was positioned at an average lateral talar bone-component (γ) angle of 15.7 degrees (range, 7.8 to 24.6 degrees) in Group A and 14.8 degrees (range, 3.6 to 23.2 degrees) in Group B. All parameters (α, β, and γ angle) showed no significant difference (p > 0.05 for each) (Table 4). Talar component subsidence or loosening did not occur in any of the cases.

### Additional procedures

Additional procedures were carried out before or at the time of total ankle arthroplasty to correct accompanying malalignment, joint contractures, or instabilities, including 17 ankles (34.0%) in group A and 15 ankles (27.8%) in group B (p = 0.354). Preoperatively, an Ilizarov correction for complex ankle deformity was performed in 2 ankles in group B. Intraoperatively, a percutaneous Achilles tendon lengthening procedure was performed 4 ankles (8.0%) in group A and 3 ankles (7.4%) in group B. Deltoid release was performed in 6 ankles (12.0%) in group A and in 4 ankles (7.4%) in group B, and a modified Broström procedure was performed 4 (8.0%) in group A and 3 (7.4%) in group B. One medial malleolar lengthening osteotomy for ligament imbalance, Dwyer osteotomy and syndesmotic fusion were performed in each group.

### Complications

Sixteen perioperative complications occurred in 11 ankles (22.0%) in group A, and 16 in 14 ankles (25.9%) in group B. There was no significant difference in terms of postoperative complications between the 2 groups (p = 0.663). One (2.0%) deep infection detected in group A at 6 months that required the changing of both tibial and talar components; the patient has had no pain and good functional results at 36 months after revision. Five (10.0%) ankles sustained an intraoperative malleolar fracture in group A and 3 (5.6%) in group B; all were non-displaced and stabilized by screw fixation, and no fracture occurred subsequently. A minor wound complication occurred in 3 (6.0%) ankles in group A and in 5 (9.3%) ankles in group B; all resolved after topical dressing changes. Two (3.7%) cases of deep peroneal neuropraxia occurred in group B, and 3 (6.0%) tendon lacerations occurred in group A (1 flexor hallucis longus and 2 extensor hallucis longus) and 1 (1.8%) in group B (flexor hallucis longus). Symptomatic heterotopic ossification was detected in 2 (4.0%) ankles in group A and 3 (5.6%) ankles in group B. Among them, 2 ankles in group B required surgical resection because of intractable pain and joint stiffness. In each group, there was a single case of medial bony impingement, both of which were managed by osteophyte resection. Tarsal tunnel release was performed on 1 ankle with tarsal tunnel syndrome in each group.

## Discussion

In most normal ankles, the bony anatomy as well as the soft-tissue structures, including joint capsule, ligaments and muscle tendon units that cross the joint, prevent significant translation of the talus relative to the mortise with positioning of the ankle [1,7,10]. These structures guide and restrain movement between the talus and the mortise so that the talus has a continuously changing axis of rotation relative to the mortise as it moves from maximum dorsiflexion to maximum plantar flexion [18]. Ankle sagittal malalignment including anterior or posterior translation of the talus relative to the tibia often occurs in patients with osteoarthritis of the ankle, [1-4] and anterior translation of the talus seems to be more common [1].

Takakura et al. [5] suggested that the talus is translated anteriorly due to stretching of the anterior talofibular ligament and chronic lateral instability is caused as a result of anterior talofibular ligament dysfunction. Thus, loading stress is concentrated on the front of the ankle and osteoarthritis develops. In addition, the amount of anterior opening of the distal tibial joint surface increases in osteoarthritis of the ankle. However, the definite mechanism of anterior translation of the talus in ankle osteoarthritis is not well understood.

Measurement of ankle sagittal alignment before and after the operation has shown that total ankle arthroplasty frequently restores an anteriorly translated talus to an anatomical position [1,2,19]. Barg et al. [19] reported that in 127 (34.5%) of 368 osteoarthritic ankles, the center of the talar component in the sagittal plane was positioned precisely in the line of the longitudinal axis of the tibia after total ankle arthroplasty, and in the other two-thirds of ankles, the variations in the talar component position were within a very small range at an average follow-up of 51.2 months. Wood et al. [2] demonstrated that 46 (33%) of 138 osteoarthritic ankles showed anterior translation of the talus, and 40 (87%) of 46 ankles showed an improvement in the alignment of the talus within the ankle mortise after total ankle arthroplasty at a minimum follow-up of 36 months. In our study, an anteriorly translated talus was detected in 50 (46.3%) of 108 osteoarthritic ankles and 48 (96.0%) of these 50 ankles restored to an anatomical position at a mean follow-up of 43 months. The authors believe that the realigned ligaments after clearance of the bony osteophytes, adequate balancing of medial and lateral ankle ligaments, anatomical positioning of the talar component, and optimal sagittal alignment may help restoration of the talus into the mortise. Moreover, Barg et al. [19] suggested that the use of conical, anatomically shaped talar surfaces, as is the case in the HINTEGRA ankle prostheses, allows for balancing of medial and lateral ankle ligaments, and thus also increases the anteroposterior stability of the replaced ankle joint. These findings support the assumption that restoration of the soft-tissue stabilizers may help realign the talus within the mortise.

However, according to the result of anteriorly translated talus group, although the talus was restored to the ankle mortise in the majority of cases at the final follow-up, they were still more anteriorly positioned than non-translated talus group. We can supposed that, in anteriorly translated talus group, soft tissue balancing was less performed and this could be the result of decreased alignment recovery ability after soft tissue imbalancing due to prolonged translated position. Because of prolonged periods of anterior translation of the talus, ability for alignment recovery might be decreased. And this could result in insufficient soft tissue balancing in anteriorly translated talus group. Therefore, we need prospective systematic study on mechanism and rate of relocation according to the severity of anterior translation of the talus.

Concerning the talar component positioning, even though the talus was translated anteriorly before performing primary total ankle arthroplasty, we implanted the talar component in an anatomical position of the native talar dome. Hintermann et al. [8] reported that positioning of the talar component too posteriorly caused talar component loosening or sagittal instability. Tochigi et al. [14] demonstrated that an anteriorly positioned talar component led to premature polyethylene bearing lift-off in the cadaver study, therefore the talar component should be placed in the anatomical position and slightly posterior placement may be acceptable if the joint height remains anatomical. Therefore, anterior placement of the talar component should be avoided.

Our results indicate that the clinical and radiographic outcomes in the osteoarthritic ankles with anteriorly translated talus group were comparable with those in non-translated talus group in terms of AOFAS score, ankle ROM, and radiographic values. We believe that restoring optimal sagittal alignment is crucial for successful total ankle arthroplasty in osteoarthritic ankles with anteriorly translated talus.

The present study has some limitations. First, it was a retrospective review of a single institutional database, and the follow-up was short. Second, the authors were unable to identify the prognostic factors for restoration of the anteriorly translated talus into the mortise. So, prospective studies involving multi-center of cases with long-term evaluation are needed. Third, tibiotalar ratio is not an absolute indicator of anterior translation of the talus. But, because of its wide use in the literature, we believe that tibiotalar ratio still offer valuable measurement of anteriorly translated talus.

## Conclusions

In most cases, an anteriorly translated talus in osteoarthritic ankles was restored to an anatomic position within 6 months after successful total ankle arthroplasty. Adequate soft tissue balancing, optimal sagittal alignment, and anatomical positioning of the talar component may help restoration of the talus into the mortise. The clinical and radiographic outcomes of three-component total ankle arthroplasty in the osteoarthritic ankles with an anteriorly translated talus group were comparable with those in the osteoarthritic ankles with a non-translated talus group at the mean follow-up of 43 months.

## Abbreviations

AOFAS: American Orthopaedic Foot and Ankle Society; ROM: Range of motion.

## Competing interests

The authors declare that they have no competing interests.

## Authors’ contributions

KBL conceived of the study and participated in its design and coordination and helped to draft the manuscript. MSK participated in its design and statistical analysis. KSP collected patient’s data ans analysis. KJC carried out statistical analysis and drafted the manuscript. AP participated in the sequence alignment and drafted the manuscript. All authors read and approved the final manuscript.

## Pre-publication history

The pre-publication history for this paper can be accessed here:

http://www.biomedcentral.com/1471-2474/14/260/prepub

## References

[B1] Wood PL Prem H Sutton C Total ankle replacement: medium-term results in 200 Scandinavian total ankle replacements J Bone Joint Surg Br 2008 90 605 609 18450626 10.1302/0301-620X.90B5.19677

[B2] Wood PL Sutton C Mishra V Suneja R A randomised, controlled trial of two mobile-bearing total ankle replacements J Bone Joint Surg Br 2009 91 69 74 19092007 10.1302/0301-620X.91B1.21346

[B3] Tochigi Y Suh JS Amendola A Pedersen DR Saltzman CL Ankle alignment on lateral radiographs. Part 1: sensitivity of measures to perturbations of ankle positioning Foot Ankle Int 2006 27 82 87 16487458 10.1177/107110070602700202 PMC2274959

[B4] Tochigi Y Suh JS Amendola A Saltzman CL Ankle alignment on lateral radiographs. Part 2: reliability and validity of measures Foot Ankle Int 2006 27 88 92 16487459 10.1177/107110070602700203 PMC2267757

[B5] Takakura Y Tanaka Y Kumai T Tamai S Low tibial osteotomy for osteoarthritis of the ankle. Results of a new operation in 18 patients J Bone Joint Surg Br 1995 77 50 54 7822395

[B6] Tanaka Y Takakura Y Hayashi K Taniguchi A Kumai T Sugimoto K Low tibial osteotomy for varus-type osteoarthritis of the ankle J Bone Joint Surg Br 2006 88 909 913 10.2106/JBJS.E.01398 16798994

[B7] Pyevich MT Saltzman CL Callaghan JJ Alvine FG Total ankle arthroplasty: a unique design. Two to twelve-year follow-up J Bone Joint Surg Am 1998 80 1410 1420 9801209

[B8] Hintermann B Valderrabano V Dereymaeker G Dick W The HINTEGRA ankle: rationale and short-term results of 122 consecutive ankles Clin Orthop Relat Res 2004 424 57 68 15241144 10.1097/01.blo.0000132462.72843.e8

[B9] Conti SF Wong YS Complications of total ankle replacement Foot Ankle Clin 2002 7 791 807 10.1016/S1083-7515(02)00050-5 12516734

[B10] Myerson MS Mroczek K Perioperative complications of total ankle arthroplasty Foot Ankle Int 2003 24 17 21 12540076 10.1177/107110070302400102

[B11] Lee KB Cho SG Hur CI Yoon TR Perioperative complications of HINTEGRA total ankle replacement: our initial 50 cases Foot Ankle Int 2008 29 978 984 10.3113/FAI.2008.0978 18851813

[B12] Barg A Elsner A Anderson AE Hintermann B The effect of three-component total ankle replacement malalignment on clinical outcome: pain relief and functional outcome in 317 consecutive patients J Bone Joint Surg Am 2011 93 1969 1978 10.2106/JBJS.J.01415 22048091

[B13] Saltzman CL Tochigi Y Rudert MJ Mclff TE Brown TD The effect of agility ankle prosthesis misalignment on the peri-ankle ligaments Clin Orthop Relat Res 2004 424 137 142 15241155 10.1097/01.blo.0000132463.80467.65

[B14] Tochigi Y Rudert MJ Brown TD McIff TE Saltzman CL The effect of accuracy of implantation on range of movement of the Scandinavian total ankle replacement J Bone Joint Surg Br 2005 87 736 740 15855381 10.1302/0301-620X.87B5.14872

[B15] Kitaoka HB Alexander IJ Adelaar RS Nunley JA Meyerson MS Sanders M Clinical rating systems for the ankle-hindfoot, midfoot, hallux, and lesser toes Foot Ankle Int 1994 15 349 353 10.1177/107110079401500701 7951968

[B16] Naal FD Impellizzeri FM Rippstein PF Which are the most frequently used outcome instruments in studies on total ankle arthroplasty? Clin Orthop Relat Res 2010 468 815 826 10.1007/s11999-009-1036-y 19672670 PMC2816756

[B17] Bai LB Lee KB Song EK Yoon TR Seon JK Total ankle arthroplasty outcome comparison for post-traumatic and primary osteoarthritis Foot Ankle Int 2010 31 1048 1056 10.3113/FAI.2010.1048 21189204

[B18] Chou LB Coughlin MT Hansen S Jr Haskell A Lundeen G Saltzman CL Mann RA Osteoarthritis of the ankle: the role of arthroplasty J Am Acad Orthop Surg 2008 16 249 259 18460685 10.5435/00124635-200805000-00003

[B19] Barg A Elsner A Chuckpaiwong B Hintermann B Insert position in three-component total ankle replacement Foot Ankle Int 2010 31 754 759 10.3113/FAI.2010.0754 20880477

